# Frailty, fragility and falls in older people: What’s new?

**DOI:** 10.1016/j.clinme.2026.100573

**Published:** 2026-03-23

**Authors:** Ponnusamy Saravanan

**Affiliations:** a Warwick Medical School, Warwick Applied Health, University of Warwick, Coventry, UK; b Centre for Global Health, University of Warwick, Coventry, UK; c Centre for Early Life, University of Warwick, Coventry, UK; d Diabetes, Endocrinology and Metabolism, George Eliot Hospital NHS Trust, Nuneaton, UK

With the global increase in ageing populations, the rates of dementia, frailty and sarcopenia have risen concurrently.^1^ Each of these conditions significantly contributes to the risk of falls, both in the community and within hospital settings. Despite advancements in our understanding of the biology of ageing, effective strategies to prevent falls and mitigate their consequences are still lacking. I am pleased to comment on three insightful articles addressing falls in the older people in this issue of *Clinical Medicine*.

Arun and Lewis^2^ beautifully explore the ‘deconditioning’ of older individuals soon after they present to the hospitals, whatever their initial presentation. This article provides a useful overview on why this happens, potential warning signs at presentation and advises the healthcare professionals to focus on ‘PINCHME’. They also highlight strategies for prevention, but an evidence-based approach is currently lacking. Of course, awareness is the first step.

Lee and Vindlacheruvu^3^ in their article summarise the multifactorial risk factors of frailty and the need for a multidisciplinary approach. They provide a practical summary on contributors and stages of frailty. For practising clinicians, this article also provides practical steps to ensure that truly multidisciplinary care is provided for older inpatients, including an updated summary for managing bone fragility.

The article by Chan summarises the current evidence base for the use of artificial intelligence (AI) in improving the care of older people with frailty and falls.^4^ With rapid advancement of AI tools in all walks of our life, including the use of AI for risk stratification in many medical disorders,^5^ this paper provides the argument for not excluding older people by citing ethical concerns and digital ageism. Dr Chan’s highlights on the current evidence base, opportunities, challenges and practical steps that healthcare professionals can adopt when encountered with AI-enabled tools. While I have no doubt that this area of medicine will rapidly evolve, these steps provide invaluable tips.

Finally, my articles of choice are:

− One by Miller *et al*,^6^ which highlights the opinions of palliative and rehabilitation specialists on management of prolonged disorders of consciousness. Nearly half of the respondents withdraw clinically assisted nutrition and hydration. In the renewed debate on assisted dying across the world, this article provides useful insight on current practice and the call for potential update of NICE guidelines, sentiments echoed recently by one of our editorials.^7^
− An educational case report by Riley *et al*^8^ on genetic aetiologies of bronchiectasis. This case highlights the need for clinicians to be vigilant of revisiting diagnosis, even several years after initial presentation. For those who missed the recent CME article on primary ciliary dyskinesia, this is a useful opportunity to relearn about this rare, inherited but potentially debilitating condition.^9^

## Funding

This research did not receive any specific grant from funding agencies in the public, commercial, or not-for-profit sectors.

## Declaration of competing interest

Prof Saravanan is the editor-in-chief of *Clinical Medicine*.

## References

[1] Sayer A.A. (2025). Harveian Oration 2024: from bench to bedside and beyond – new horizons for translational ageing research. Clin Med.

[2] Arun B., Lewis S.H.M. (2026). Frailty and deconditioning on the acute take. Clin Med.

[3] Lee C., Vindlacheruvu M. (2026). Bridging geriatrics and trauma: evidence-based care for falls in older adults. Clin Med.

[4] Chan C.P.I. (2026). Using data and artificial intelligence to improve care pathways of older people experiencing falls and frailty: opportunities, challenges and practical considerations for clinicians. Clin Med.

[5] Kumar K., Saravanan P. (2025). Population-specific risk models and AI in clinical practice: are we ready for the next step in managing common disorders?. Clin Med.

[6] Miller M., Harrison T., Barrie S., Pick A. (2026). Prolonged disorders of consciousness: practice described by palliative and rehabilitation physicians. Clin Med.

[7] Taylor A., Davies A., Saravanan P. (2025). The changing landscape of palliative care: a universal imperative. Clin Med.

[8] Riley D., Kumar K., Stowell S.M. (2026). Genetic aetiologies of bronchiectasis revisited: a new diagnosis of primary ciliary dyskinesia in adulthood. Clin Med.

[9] Collison R., Hyatali S.A., Kamenova A. (2025). Primary ciliary dyskinesia: aetiology, diagnosis and clinical management. Clin Med.

